# Delivering scalable Telehealth: ‘What is Scale?’ with case studies from NHS providers, a perspective on the challenges, constraints and issues associated with ‘scalability’

**Published:** 2012-06-15

**Authors:** Sonia Jane Milburn, Adrian Flowerday

**Affiliations:** Solent NHS Trust, United Kingdom; Docobo Limited, United Kingdom

**Keywords:** scalability, success, implementation, scalable, rollout

## Abstract

**Introduction:**

With local and national evidence highlighting and confirming numerous benefits gained from implementing telehealth, why is it so hard to roll out the technology at scale? Solent NHS Trust has worked alongside Docobo Limited, since 2005, to successfully introduce telehealth to its patients and clinical staff. Our Chronic Obstructive Pulmonary Disease, Chronic Heart Failure and Community Matron teams have utilised Telehealth tools into their normal practice and have delivered significant benefits, such as improved patient education, improved case loads and reduced hospital admissions. As a result we have developed a unique joint perspective on the success factors that address the challenges, constraints and issues faced by both the telehealth supplier and NHS provider when rolling out this technology. Our joint experience will highlight factors and areas (some obvious and some ‘not so obvious’) that may act as blockers to rolling out Telehealth technology at scale. We will provide some suggestions on addressing these issues alongside some tips for success.

**Aims:**

The aim of this presentation is to inform delegates of factors that need to be considered when trying to achieve a scalable rollout of Telehealth alongside delivering some tips for success. Areas to be discussed during the presentation include:

- Identifying the right patients—patient stratification
- Installation capacity and scalable support infrastructure
- Cultural change—attitudes and beliefs
- Implementation in a changing business environment
- Educating the wider healthcare community
- Avoiding units gathering dust on shelves.

**Conclusions:**

The talk will conclude with the following summarisations:

- Small scale local implementation creates the foundation for a wider deployment
- Small implementations help identify key issues and problems that will need to be addressed to achieve scalability
- Need to accept that implementation is a continual process of development and learning that creates opportunities
- Advantages in having a consistent relationship with the delivery partner and that as technology evolves and business develops the technology matches customer requirements
- Persistence in developing the telehealth agenda creates the opportunity for wider deployment—essential to start small but have a big vision
- Essential to use Risk Stratification dashboards to identify savings when rolling out Telehealth into mainstream operational practice—the only way to identify savings generated by the intervention.

